# Qualitative insights into the experiences and goals of people living with severe difficult-to-treat hypertension in Australia

**DOI:** 10.1186/s12872-026-06141-9

**Published:** 2026-06-19

**Authors:** Michelle Hill, Jenni Godsell

**Affiliations:** 1 Medtronic Australasia, Macquarie Park, NSW Australia; 2 Community and Patient Preference Research Pty Ltd, PO Box 2214, Woolooware, NSW 2230 Australia

**Keywords:** Blood pressure, Renal denervation, Patient participation, Severe difficult-to-treat hypertension, Qualitative research, Interview

## Abstract

**Background:**

Over one-third of Australian adults have hypertension, and this rate increases with age. Difficult-to-treat hypertension is high blood pressure that remains above target levels despite proper medicine adherence. This study explored the treatment experiences and goals of patients with severe difficult-to-treat hypertension to gain insights into renal denervation (RDN) as a therapeutic option.

**Methods:**

Twelve Australian participants with severe difficult-to-treat hypertension were interviewed. Participants were recruited via a specialist recruitment company and specialist referral. Semi-structured interviews lasted about 60 min either online or by phone. Interviews were recorded and transcribed verbatim. Transcripts were analyzed using inductive and deductive thematic analysis in NVivo.

**Results:**

Participants had an average age of 65 years (range: 48–75 years), 58% identified as male and all lived across Australia, primarily in metropolitan areas (*n* = 9; 75%). The average time since hypertension diagnosis was 22 years (range: 3–44 years) and nearly all participants had a family history of hypertension. Data analysis identified four themes regarding participants’ experiences of living with severe difficult-to-treat hypertension and awareness of renal denervation therapy: (1) Diagnosis and monitoring of hypertension, (2) The impact of severe difficult-to-treat hypertension on quality of life, (3) The management of severe difficult-to-treat hypertension and (4) Awareness, perceptions and experiences with RDN.

**Conclusions:**

This study provides an in-depth understanding of the experiences of people living with severe difficult-to-treat hypertension and their treatment goals. It highlights the significant impact and stigma of living with severe difficult-to-treat hypertension, and the need to incorporate psychosocial support and patient education into the clinical management of this chronic condition.

**Supplementary Information:**

The online version contains supplementary material available at https://doi.org/10.1186/s12872-026-06141-9.

## Introduction

One in three Australian adults have elevated blood pressure (hypertension) and this rate increases with age [1]. Blood pressure cut-off values aid in the diagnosis and management of hypertension (Table 1) [2]. If left untreated, hypertension can lead to kidney disease, heart disease, heart failure, and stroke [3].

**Table 1:** Classification of clinic blood pressure levels in adults [2]

Diagnostic category	Systolic (mmHg)		Diastolic (mmHg)
Optimal	< 120	and	< 80
Normal	120–129	and/or	80–84
High-normal	130–139	and/or	85–89
Grade 1 (mild) hypertension	140–159	and/or	90–99
Grade 2 (moderate) hypertension	160–179	and/or	100–109
Grade 3 (severe) hypertension	≥ 180	and/or	≥ 110
Isolated systolic hypertension	> 140	and	< 90

The current Australian Hypertension Clinical Guidelines recommend starting with a low-moderate dose of a first-line antihypertensive medication. If the treatment is poorly tolerated, it can be changed to a different drug class, starting at a low-moderate dose [2]. If the target blood pressure is not reached after three months, it is recommended to add a second drug, from a different pharmacological class, at a low-moderate dose [2]. The aim is to maximize drug efficacy while minimizing adverse effects [2]. If the target blood pressure is still not reached after an additional three months, it is recommended to incrementally increase the dosage of one drug, provided that both drugs are well tolerated. If the maximum dosage of one drug is reached, then the dosage of the second drug may be incrementally increased. If the target blood pressure is still not reached after three months, despite a maximal dose of at least two drugs, initiation of a third drug class at a low-moderate dose may be considered. If the patient’s blood pressure remains elevated, it is recommended that they be referred to a specialist [2].

Treatment-resistant hypertension occurs when blood pressure remains over target levels despite adherence to guideline-recommended optimal medical management with antihypertensive drugs. It is defined as systolic blood pressure of ≥ 140 mmHg in a patient taking three or more antihypertensive drugs of different classes, including a diuretic, at optimal tolerated doses [2].

A related but broader term, difficult-to-treat hypertension refers to patients whose blood pressure remains uncontrolled despite the use of multiple antihypertensive medications, but who may not meet the strict criteria for treatment-resistant hypertension. This can occur, for example, when a diuretic is not included in their regimen due to intolerance or other clinical considerations.

Common treatment options for difficult-to-treat hypertension include combinations of different classes of medication including beta blockers (BBs), calcium channel blockers (CCBs), angiotensin-converting enzyme (ACE) inhibitors, and angiotensin receptor blockers (ARBs) along with diuretics. Patients may undergo renal denervation (RDN) to address the underlying causes of their uncontrolled blood pressure [2]. RDN disrupts the neurogenic reflex involved in blood pressure control, using a catheter-based technique under local anesthesia [2]. Long-term follow-up shows RDN results in a clinically meaningful reduction in both systolic and diastolic blood pressure, less medication, and no adverse consequences on renal function [4]. While RDN appears promising for patients with difficult-to-treat hypertension, particularly those with multiple medication intolerances, evidence on its long-term benefits is still emerging [5]. The patients’ experiences of living with difficult-to-treat hypertension and their views on the available treatment options have not been fully explored in the Australian context. Additionally, as RDN is an emerging treatment option, it is important to understand and assess patients’ interest in it. Given the limited patient knowledge of RDN, this study aimed to gain an understanding from patients on the impact of severe difficult-to-treat hypertension on their lives and their knowledge and perceptions of RDN as a therapeutic option. A secondary objective was to explore firsthand experiences of patients who had undergone RDN as treatment for their hypertension.

## Methods

### Study design

To be eligible for an interview, participants had to be aged 18 years or older; have severe difficult-to-treat hypertension that was considered to represent a ‘high clinical need’. For this study ‘high clinical need’ was defined as having a systolic blood pressure reading of ≥ 180 mmHg and/or a diastolic blood pressure reading of ≥ 120 mmHg (based on the American Heart Association guidelines) and be currently taking three or more antihypertensive medications as individual or combination therapy (a diuretic was not a requirement for inclusion); be an Australian citizen or permanent resident; and provide written informed consent. Participants who had undergone RDN were identified by physician referral, must have taken three or more antihypertensive medications as individual or combination therapy at some point in their treatment journey and had RDN within the last 3 months to 5 years. This timeframe was chosen because it was considered sufficient time to see the effects of RDN and a reasonable time where participants would still remember the procedure. The inclusion was not limited to any specific RDN device, and the device used in each participant’s procedure was not recorded as part of the study data. Regarding the dynamic nature of hypertension [6], participants who had previously experienced severe uncontrolled hypertension, but whose blood pressure was controlled at the time of the study were included. Participants were excluded if employed by a pharmaceutical, medical device, or vaccine company.

Participants were recruited via a specialist recruitment company and specialist referral of eligible patients to the study team. Eligible patients were contacted by email or telephone. Participants were informed during the recruitment process that the research would involve discussing the impact of living with severe difficult-to-treat hypertension and their experiences with treatment. After completing the interview, participants received an electronic gift card for AUD$120 in acknowledgment of their time.

### Interviews

The interviews were conducted by Zoom video conferencing or by telephone by JG (BA (Psychology), Post Grad Dip (Psychology), a female with approximately 20 years’ experience in conducting focus groups and in-depth interviews for healthcare research. JG did not know or have a professional relationship with any of the participants prior to the interviews. The interviews lasted between 45 and 60 min and were conducted between August 22 and September 17, 2024. In order to mitigate potential bias, the participants were unaware that the research was conducted for Medtronic until disclosure by the researcher directly after the interview. Non-RDN participants continued to be recruited and interviewed until data saturation was reached where no new knowledge was being obtained (*n* = 10). Two participants who had previously undergone RDN were purposely recruited and interviewed to gain insight into their lived experiences, including impact on quality of life. This information was viewed as supportive to the broader objective of understanding perceived value of RDN among participants who only had experience with antihypertensive medication.

### Data collection

Semi-structured interviews were conducted using a discussion guide, which is provided in Additional file 1. This approach was taken to ensure that all research objectives were met. The discussion guide covered five areas:

1. Participant demographics.
2. Impact of severe difficult-to-treat hypertension.
3. Management of severe difficult-to-treat hypertension, including experience with antihypertensives.
4. Treatment goals.
5. Awareness, perceptions, and experiences with RDN.

Participants who were not aware of RDN were provided with a short description of the procedure (Additional file 1). The interviews were recorded on Zoom and transcribed verbatim by an independent transcriber. Field notes were taken during the interviews to support analysis. Interview transcripts were not returned to participants for their comments, as such feedback was not required for the study objectives. Additionally, interviewee transcript review can affect the accuracy of candid responses and add additional burden to the participant [7].

### Ethical considerations

Formal ethics approval was not required for this study. The research was assessed as having a low level of risk and met the definition of negligible risk research, qualifying for exemption from ethical review, as defined in the National Statement on Ethical Conduct in Human Research Chap. 2.1 and Chap. 5.1.17 [8]. This meets the assessment requirements of Bellberry Human Research Ethics Committee [9].

All participants were required to read an information sheet outlining data use, confidentiality, and the benefits and risks of participation. All participants provided written consent before taking part in the research. This study complied with the Declaration of Helsinki [10].

### Analysis

Interview transcripts were analyzed using inductive and deductive thematic analysis [11, 12]. Thematic analysis places emphasis on the identification, organization, analysis and interpretation of themes (i.e., patterns of meaning) and is therefore an appropriate approach when determining participant views, knowledge, experiences or values [13]. The inductive process encouraged the analysis of themes directly from the transcripts. The deductive approach was developed to include themes that were directly in line with the study objectives [12].

Initially, transcripts were reviewed by a single researcher (JG, CaPPRe) for an overview of participant perceptions. The qualitative code frame was generated by JG (lead analyst) and subsequently refined and grouped to form initial themes. A comprehensive framework was developed that enabled systematic analysis of the data leading to the evaluation and interpretation of conclusions and study insights. To support validity and reliability of the code frame and study insights, findings were independently reviewed by a second researcher (MJ, CaPPRe). Data analysis occurred concurrently with data collection.

The coding of interview data was an iterative process, and ongoing refinements were made based on further examination of the transcripts. The interview transcripts were analyzed using NVivo™ v12 (Lumivero, Denver, CO, USA). The study is reported in line with the consolidated criteria for reporting qualitative research checklist for interviews and focus groups (Additional file 1) [14].

### Reflexivity

In order to enhance transparency and uphold the scientific rigor of the study, reflexivity was systematically addressed throughout the study process. The researchers’ professional backgrounds and experience in qualitative research were considered at each phase of the study to identify any potential influence on data collection and interpretation. Furthermore, the researchers engaged in regular peer debriefing sessions to review coding decisions and to validate theme development throughout the data analysis.

## Results

### Participants demographics

Twelve participants met the study inclusion criteria and were included in the study (Additional file 2). Participants had an average age of 65 years (range: 48–75 years) and 58% identified as male (Table 2). Participants reported living across Australia (Victoria *n* = 5, Western Australia *n* = 3, New South Wales *n* = 3 and Australian Capital Territory *n* = 1) and primarily lived in metropolitan areas (*n* = 9; 75%). The approximate average time since diagnosis was 22 years (range: 3–44 years).

**Table 2:** Demographics and other baseline characteristics

Characteristic	Number of respondents (*n* = 12)
Mean age, years (range)	65 (48–75)
Sex:	
Male	7
Female	5
State or territory of residence:	
Victoria	5
Western Australia	3
New South Wales	3
Australian Capital Territory	1
Geographical area:	
Metropolitan	9
Non-metropolitan	3
Time since diagnosis, years (range)	22 (3–44)
Previous RDN treatment:	
Yes	2
No	10
Current medication classes used by participants:	
Alpha blockers	4
ACE inhibitors or ARBs	12
BBs	6
CCBs	10
Diuretics	7
Use of a combination products containing medication from different drug classes:	6
Combination products that included a diuretic	4

*n * number of cases, *RDN * renal denervation

All non-RDN participants were currently on three or more medications for hypertension, which included a mixture of individual products or use of a combination product containing medication from different drug classes (*n* = 6). Key classes of medications being used were ACE inhibitors or ARBs (*n* = 12), CCBs (*n* = 10), diuretics (*n* = 7), BBs (*n* = 6) and alpha blockers (*n* = 4).

### Medical history

Nearly all participants spoke of a family history of hypertension; some had relatives who had died from heart failure. Participant 9 revealed that they had a *“Huge family history. My mum had high blood pressure*,* and she was being treated for it*,* her brothers had cardiovascular disease and hence died from that.*” Participants also spoke about also living with comorbid conditions, such as Type 2 diabetes, mental health disorders and cardiovascular diseases. Some participants perceived their current level of health to be low, due to persistent symptoms of hypertension. When asked about their medical history, Participant 1 said *“Well*,* this…atrial fibrillation has got worse*,* and my mitral valve has got worse…which does increase my blood pressure even though I’m on medication.”*

#### Themes

**Fig. 1:**
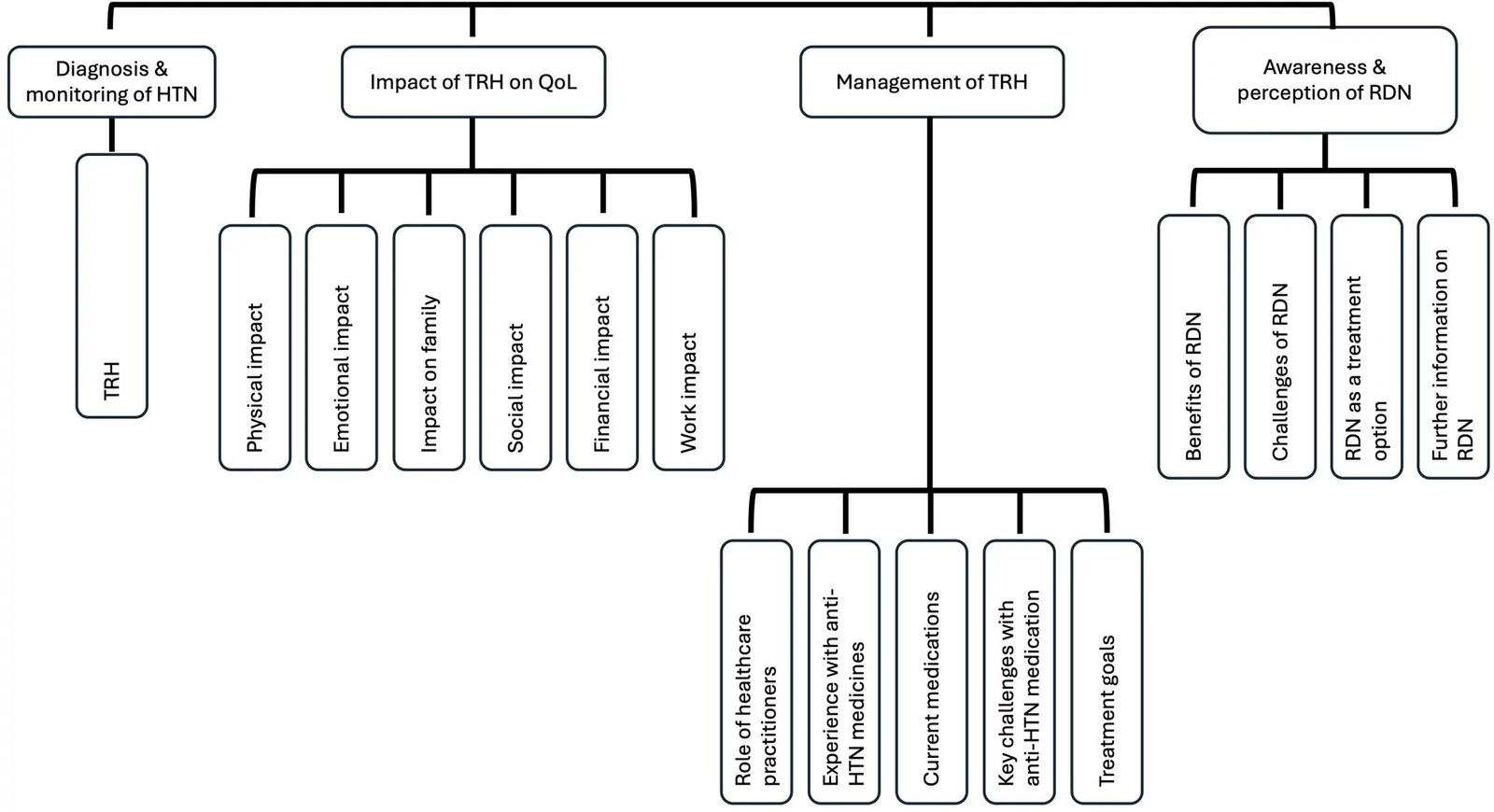
Coding tree. HTN = hypertension; DTH = difficult-to-treat hypertension; QoL = quality of life; RDN = renal denervation

Data analysis identified four themes: (1) Diagnosis and monitoring of hypertension, (2) The impact of severe difficult-to-treat hypertension on quality of life, (3) The management of severe difficult-to-treat hypertension and (4) Awareness, perceptions and experiences with RDN (Fig. 1).

### Diagnosis and monitoring of hypertension

Some participants had been diagnosed with hypertension incidentally. Others received their diagnosis after experiencing symptoms or monitoring their blood pressure for other conditions (Additional file 3). All participants owned a blood pressure monitor and spoke of regularly monitoring their blood pressure. Some also kept a record of their blood pressure readings to monitor their condition (Additional file 3).

### Severe difficult-to-treat hypertension

While participants were generally unfamiliar with clinical terminology for difficult-to-treat hypertension (e.g. persistent hypertension, treatment resistance), all acknowledged that they were living with high blood pressure that was difficult to control and subject to fluctuations (Additional file 3). They spoke of trying numerous combinations of antihypertensive medications. However, all participants had at some point experienced alarmingly high blood pressure readings. A few participants attributed spikes in their blood pressure to stress, anxiety and other events or health conditions.

### Impact of severe difficult-to-treat hypertension on quality of life

#### Physical impact

Several participants experienced ongoing symptoms that, for some, resulted in visits to the emergency department. Three participants had visited the hospital specifically for their severe difficult-to-treat hypertension, due to spikes in their blood pressure readings. Several participants reported medication causing low blood pressure and hypotensive symptoms.

### Emotional impact

Participants were acutely aware of the implications of uncontrolled blood pressure, particularly the risk of premature death from heart attack and stroke (Additional file 3). Several individuals expressed anxiety and for some, this had exacerbated their hypertension. All participants reported high blood pressure readings which had caused them significant concern. Participant 2 said *“I know they call it the silent killer. I’ve heard that expression before and I think the reason…is the fact*,* it doesn’t make you feel sick.”*

One individual who had experienced significant problems with elevated blood pressure while undergoing a general anesthetic for surgery, expressed concern regarding any future surgery.

Fear was voiced by two individuals who lived alone, in case a critical situation should occur (Additional file 3). Two participants also felt some stigma in living with hypertension and having to take medication. Participant 6 noted *“I think an element of being a little bit self-conscious earlier on when I was younger*,* because it’s quite young to be having blood pressure medication. You tend to see this in quite overweight and older people.”* One participant appreciated being able to take medication discreetly due to feeling there was a stigma in living with hypertension.

#### Impact on family

Most participants had not had to rely on support from a carer or loved one, although several spoke appreciatively of their spouses and other family members who provided help when required. A few participants spoke of the importance of emotional support from their family, and the concern felt by family members, particularly about the risks associated with hypertension. One participant mentioned the inheritable nature of the disease (Additional file 3).

#### Social impact

Some participants mentioned limiting their social activities due to their symptoms, which had precluded them from engaging in activities, such as bush walking, visiting theme parks and traveling. When asked about the social impact of living with severe difficult-to-treat hypertension, Participant 8 said *“It got to a stage where I just couldn’t socialize for any length of time*,* I used to get tired very quickly and everything was just too much of an effort.”* Several participants mentioned changing their lifestyle due to their condition (Additional file 3). A few participants also mentioned that side effects of their medications had caused some social restrictions.

#### Financial impact

Overall, participants had not experienced significant out-of-pocket costs for the treatment and management of their hypertension. This was attributed to the low cost of medication which was noted as being more expensive in the past by one participant who had been living with hypertension for approximately 40 years. When asked about the financial impact, participant 4 said *“To begin with there wasn’t much change out of $100. Now I can get all my medications for under $30.”* There was some acknowledgment of significant long-term expenses in living with severe difficult-to-treat hypertension for many years (Additional file 3). Some participants mentioned that they had kept costs down by being on the pension, having a Commonwealth health card, having private health insurance or being bulk billed by their health care practitioner (HCP). However, a few participants had still reached the Medicare safety nets. The Medicare safety nets give more money back to patients who spend over a certain amount on medical expenses [15].

#### Work impact

Generally, participants’ participation in work had not been impacted by their hypertension. There had been some minor effects such as taking sick leave or leaving work early occasionally. One individual had to stop a job as a musician due to their symptoms. Two participants spoke about their decreased work participation due to poor health caused by other health issues.

### Management of severe difficult-to-treat hypertension

#### Role of health care practitioners

Participants reported that their general practitioners (GPs) were key in managing their condition. Most saw their GP regularly (i.e., every three months) for ongoing monitoring. GPs adjusted medication doses or switched medications depending on patient feedback and/or patient records of their blood pressure readings. They also arranged blood tests as required. Generally, participants felt involved in treatment decisions, however they felt that key treatment responsibilities lay with their HCP (GP or specialist), whom they trusted (Additional file 3). A few participants were also regularly seeing a specialist once or twice a year for management of other conditions. Other participants had attended appointments with a cardiologist or seen a cardiologist in hospital.

#### Experiences with antihypertensive medication

Nearly all participants commenced an antihypertensive medication upon diagnosis. One individual was monitored for a few years, to try to improve their blood pressure through diet and exercise, before medication initiation.

Participants noted that their satisfaction with medication had varied, particularly during times of resistance, where their blood pressure had become unstable and difficult to control. Many participants had tried several different medications, noting that there had been much ‘trial and error’ in finding a combination that offered long-term, effective control.

Participants were appreciative of medication they had tried, in terms of keeping them sufficiently controlled and protected from experiencing a heart attack or stroke.

They envisaged being worse off without medication. However, in meeting goals in life, several participants had higher expectations as their medication had not been successful in reducing their blood pressure to a consistently stable and safe level, leading to significant restrictions in physical and social activities. Several participants experienced anxiety related to the potential complications of their hypertension, restricting pursuit of goals.

#### Current medications

Participants reported having many medication changes over time including dose changes and switching of medicines. Some participants mentioned commencing with one tablet upon diagnosis and subsequently having further medicines added due to insufficient hypertension control. This ranged in time frame, with some staying on one tablet for several years (although some participants had trouble remembering the details). Others were initiated on a combination of tablets at diagnosis. Examples of trialing between 6 and 20 antihypertensives prior to their current regimen were given. However, the need to take ongoing medication also had an emotional impact with participants concerned about potential long-term impacts. Participant 2, felt their medication impacted on them emotionally, referring to it “*as a nuisance because having to take so many different medications…it’s a bit disheartening.”*

#### Key benefits and challenges with antihypertensive medication

Participants expressed gratitude for their medications, especially for stabilizing their blood pressure (Additional file 3) and providing protection against stroke or heart attack. This has led to psychological benefits for several participants. For example, Participant 10 noted *“For me it’s more mental*,* it’s peace of mind…It gives me some sort of control and that’s important to me.”*

Several participants acknowledged that medication had not been effective in keeping their hypertension consistently stable. They expressed concern regarding their resistance, and how they would respond to future treatment options, if required (Additional file 3). Some participants mentioned experiencing side effects with medication (Additional file 3). These included headaches, nausea, swelling in their ankles and fingers, dizziness, visual disturbances, fatigue, bruising, weight gain, increased urinary frequency (attributed to diuretic) and anxiety. Participants spoke of switching medications due to these side effects.

#### Medication adherence

Overall, participants understood the importance of taking their antihypertensives as prescribed. Several participants mentioned that their HCP had reinforced the importance of taking their medication as prescribed (Additional file 3). Most participants reported compliance with medication as recommended. Some participants mentioned using medicine packs such as a Webster-pak, which made it easier for them to remember to take their medicines. However, most participants reported occasionally missing doses (Additional file 3). This usually occurred when their routines were disrupted.

One participant envisaged becoming more forgetful with increasing age (Additional file 3). This was echoed by another individual (Participant 3), who had found it harder to keep on top of his medication requirements due to older age and poorer health seen with *“I’ve actually got much more forgetful*,* so I’ve had a few strokes*,* and I do set alarms on my phone…I’m pretty good in the mornings*,* but if my routine is out of kilter […] I really struggle with that.”*

One individual reported occasionally titrating their medication in response to elevated blood pressure readings by adding tablets that were left over from previous prescriptions. Participant 1 explained *“I mean even this morning I got up over 200 and I thought*,* ‘Oh my gosh*,* here we go.’ I personally increased my medication this morning.”*

#### Medication load

Several participants were concerned about the number of medications they were required to take (Additional file 3). Some felt hindered by the number of antihypertensive medications required daily. They spoke of the negative impact on emotional (i.e., anxiety and feeling ‘unhealthy’) and physical wellbeing (e.g., side effects such as dizziness, urinary frequency), and to a lesser extent, minor issues with adherence (i.e., forgetting to take medication). Participants taking a combination medicine for their hypertension experienced less burden with medication load. They found it more convenient only taking one tablet a day as Participant 6 said *“I think having medicine where you’ve got three in one rather than having to take three different kinds*,* that certainly helps.”*

Other participants reported having become used to taking a lot of medication and accepted it as part of their daily routine (Additional file 3). A few participants described their hypertension as a condition that made them feel ‘old’ which was reinforced by the number of tablets required. Along with antihypertensives, several participants reported taking medication for comorbid conditions, with some also taking supplements such as krill oil and liver health tablets, which contributed to a high medication load.

#### Treatment goals

Participants’ treatment goals focused on improving their quality of life and finding treatments that were effective in keeping their blood pressure stable. Effective treatment equated to greater confidence in pursuing daily activities, with several participants desiring to engage in more exercise and hobbies without the fear of complications.

Participants spoke of the importance of avoiding premature death, as they wished to spend time with their family, which included seeing grandchildren grow up. They also did not want their family to have ongoing concerns for them and their condition. Participants spoke of improving their emotional wellbeing by having treatment that provides peace of mind. From a social perspective, this would allow people to be more confident in doing things like going out to a restaurant and having a drink.

#### Treatment goal – reducing medication load

Most participants would welcome being able to reduce their medication load, especially those who were taking antihypertensives from different classes, and for comorbid conditions. Some participants felt that reducing their antihypertensive load would lead to an improvement in psychological wellbeing, as medication has negative connotations in leading a healthy life (Additional file 3). Several participants were concerned about developing long-term side effects with medication and felt that reducing medication load would be beneficial. Participant 1 had concerns about long-term side effects as they said, *“Like my liver functions or kidney functions might come back a bit*,* not on the level that it should be […] But then you think*,* well what else are these tablets doing to the rest of my body?”*

## Awareness and perceptions of renal denervation

Two participants had undergone RDN treatment for their hypertension (Table 3). The remaining participants were not aware of the procedure or what it involved. RDN had not been mentioned as a treatment option by their HCPs (Additional file 3). Overall, participants welcomed RDN as an innovative treatment option for their hypertension. They saw several benefits with RDN and expressed a strong need to have this option available in Australia.

**Table 3:** Case study - experiences with renal denervation

Experiences with renal denervation	
Two participants who had received RDN provided insight into their experience of RDN as a therapeutic option for severe difficult-to-treat hypertension.	
Access to RDN	
One participant spoke to their cardiologist after becoming aware of RDN through social media. They were included in an RDN experimental trial in November 2020. The other participant received the procedure in November 2023, on the recommendation of their cardiologist.	
The RDN procedure	
Both participants felt that the procedure was simple and quick, taking less than half an hour.*“[It was] a morning in hospital under a local anesthetic. The procedure was using a catheter through the groin. There was no discomfort during the procedure. From a patient’s point of view the procedure was simple and painless.” Participant 12*The procedure did not incur costs, nor did the participants report side effects or any challenges following the procedure.	
Outcomes of RDN	
Both participants had experienced consistent lowering of blood pressure and felt that RDN had been more effective than medication. One participant felt that if the procedure had been available earlier, it would have alleviated the significant burden of living with severe difficult-to-treat hypertension over several decades.*“My blood pressure has come down*,* so I am more than happy. I’ve had high blood pressure for years. It’s the only thing that’s worked for me…I feel that it is very important. If it had been available 40 years ago*,* I could have had the treatment then*,* resolving the problem while I was still young.” Participant 12*The reduction in blood pressure had led to significant benefits for both participants who spoke of the physical and emotional advantages.*“I feel normal*,* I can have a pretty active lifestyle*,* doing work etc.” Participant 11“Physically I feel better and emotionally I am more conscious that I am at a reduced risk of having a heart attack or stroke. […] It’s eased my mind…it’s like there’s light at the end of the tunnel.” Participant 12*	
Treatment goals	
For one participant, RDN had reduced the number of medications from four to three, a few months after the procedure. This participant ultimately hoped to be able to reduce his antihypertensive medication load to zero.While the other participant’s blood pressure was now under control, he was hopeful that he will be able to reduce his current medications in the future. However, his key treatment goals centered on increasing his fitness and being able to travel with his family which, in turn, are likely to enhance his emotional wellbeing.*“I hope to reduce my meds but ultimately it will enable me to get fitter which will help reduce my depression and enjoy more of my life*,* and travel more with my wife.” Participant 12*	
Ongoing monitoring	
Both participants were under the regular care of a cardiologist within a hypertension clinic.	
Conclusion	
Both participants were highly satisfied with RDN and reported improvements in blood pressure control and quality of life following RDN. They were of the opinion that RDN was a life-saving treatment.*“It may just save some lives.” Participant 11*It is important to acknowledge that these findings are based on two individuals and should be interpreted with caution.	

### Perceived benefits of renal denervation

Participants indicated that they would welcome access to RDN as this procedure was linked with improved efficacy in terms of better control of blood pressure, therefore reducing the risk of life-threatening complications. As such, RDN could lead to improvements in quality of life, where participants would feel more confident in embracing activities of daily living (Additional file 3). Several participants believed that RDN would be helpful in improving their emotional wellbeing (Additional file 3). Participants would welcome the potential of RDN to lower their medication load (Additional file 3). This reduced medication load was associated with minimizing side effects and medication adherence issues. RDN was also perceived as having the benefit of reducing the costs of medication (Additional file 3). It also appealed to participants due to the convenience of a single procedure that can address issues caused by hypertension.

### Perceived challenges of renal denervation

The risk of surgery was perceived to be a potential challenge, despite the description stating that it is a minimally invasive procedure. Some participants noted that all surgical procedures carry certain risks (Additional file 3), and RDN was more invasive than oral medication. The process of targeting specific nerves with ultrasound or radiofrequency energy raised alarm for some individuals, who linked this process to a potential risk of damaging the kidneys. Other participants believed the procedure was experimental and contemplated the long-term impacts of ‘tampering’ with the body. Concern was raised by one participant (Participant 6) who felt that limiting overactive nerves near the kidneys, may trigger other conditions, or lead to underactive nerves and symptoms of hypotension. They were worried that *“modifying the body’s cells by stimulating them*,* could, after a period of time*,* [be] seen as…it causes cancer”* and *“in 20…or 15 years’ time*,* your blood pressure will come back and then it will be untreatable. You will be resistant to that therapy*,* and it will be resistant to medicine too.”*

One participant felt that unless RDN could prevent the need for medication altogether, there was not a clear advantage in being able to reduce the number, or dose of medications (Additional file 3).

### Renal denervation as a treatment option

Most participants (*n* = 6) expressed enthusiasm and a willingness to have the procedure, especially if recommended by their doctor. Two participants, whose blood pressure was well controlled on their current medication, felt that they would explore RDN as a treatment option should their circumstances change. They further stated that it was a good option for people experiencing greater resistance to medication. The participants also displayed some hesitancy due to the potential need to still take medication. They perceived RDN to be an additional treatment on top of what they were already doing. At this stage, two participants did not believe RDN would be a suitable treatment for them, due to the need for a hospital stay, which was unappealing. These individuals had already undergone multiple surgeries for other conditions and were anxious at the thought of additional surgery (Additional file 3). When asked how RDN may meet their treatment goals, participants were hopeful it would be more effective in providing stable, healthy blood pressure control, and in reducing medication load (Additional file 3).

### Further information on renal denervation

Several participants were interested in understanding more about RDN. Providing information could alleviate some concerns regarding this procedure. This information should cover how long the procedure has been available, the therapeutic outcomes of RDN and the level of expertise in conducting the procedure. Reassurance needs to be provided that RDN is not in an experimental stage (Additional file 3). Participants also wanted further information about potential side effects and risks. Specific details relating to the procedure itself, such as whether a general or local anesthetic was required, were important to a small number of participants. Participants spoke of needing more evidence of RDN’s capacity to reduce medication load (Additional file 3).

There was also request for information regarding whether Medicare covers the costs of RDN. However, again, if presented with sufficient information they would not rule out the treatment in the future (Additional file 3).

## Discussion

In this study, we presented the lived experiences of patients with severe difficult-to-treat hypertension, including their long-term treatment goals. Participants in this study were considered as having severe difficult-to-treat hypertension, as all required multiple antihypertensive medications to manage their blood pressure, although not all participants’ treatment regimens currently included a diuretic. Subsequent analysis of the interview transcripts identified four common themes:

- Diagnosis and monitoring of hypertension.
- Impact of severe difficult-to-treat hypertension on quality of life.
- Management of severe difficult-to-treat hypertension.
- Awareness and perceptions of renal denervation.

### Diagnosis and monitoring of hypertension

Participants in this study were diagnosed with hypertension and monitored through methods consistent with those previously reported in the literature. Patients were diagnosed based on symptoms or via blood pressure monitoring [16–18], with ongoing monitoring undertaken through home-based blood pressure monitors, and regular visits to healthcare professionals [19, 20]. This suggests that the diagnosis and monitoring of hypertension in Australia aligns with global practices.

### Impact of severe difficult-to-treat hypertension

Our study identified the emotional burden experienced by participants with uncontrolled blood pressure as a result of their awareness that they are at increased risk of premature death. These findings echo those from patients globally with hypertension who describe experiencing anxiety, anger, sadness and uncertainty regarding their future and the potential impact on their loved ones [21–24]. While participants’ work had not generally been impacted by their diagnosis in our study, some participants mentioned that symptoms of severe difficult-to-treat hypertension caused social restrictions. Severe difficult-to-treat hypertension has been shown to negatively impact quality of life due to many reasons such as awareness of disease, symptoms, treatments and frustrations with monitoring [21]. For many patients, the impact on quality of life is likely multifactorial [21]. Considering the significant physical and mental impact of this diagnosis, it is essential that physicians incorporate quality of life considerations during management of these patients. This study also reinforced the sense of stigma experienced by some patients, particularly those diagnosed at a younger age, appearing to stem from the perception of hypertension usually being a condition of the elderly or overweight. Stigma can evidently affect willingness to disclose the diagnosis and engage openly with treatment which can represent a discrete barrier to care [25]. HCPs should be aware that social normalization of the diagnosis may be an important, yet underappreciated component of patient support. Furthermore, at least one participant reported self-adjusting their antihypertensive medication in response to alarming home blood pressure readings, without clinical guidance. This behavior, while understandable given the patient’s distress, carries patient safety implications and highlights a gap in structured patient education around acute blood pressure events. These findings suggest that clinical management of severe difficult-to-treat hypertension should extend beyond medication optimization to include psychosocial support and clear guidance on appropriate self-management between consultations.

### Management of severe difficult-to-treat hypertension

We reported that nearly all participants commenced an antihypertensive medication upon diagnosis and reported generally good medication compliance. This finding contradicts the literature, where a large proportion of patients with difficult-to-treat hypertension are reported to be non-adherent to their medications [26]. This difference in our study, may be due to participants’ self-reported adherence and consequently, self-selection bias. As a result, caution is needed when interpreting self-reported adherence data. Although the precise causes of treatment non-adherence are likely due to a combination of different factors, evidence suggests that a high medication burden is associated with non-adherence in difficult-to-treat hypertension [26]. Despite good medication adherence in our study, participants were unhappy with the quantity of medications they were prescribed, particularly those taking tablets from different classes and for other conditions. This finding is supported by a meta-synthesis of qualitative studies examining medication burden in patients, which indicated that the management of medication regimens required significant time, money and effort; while some patients could easily incorporate this into their daily routines, others found it very difficult [27]. Additionally, some participants were concerned about developing long-term side effects from their medication. Experiencing side effects from medication can negatively impact a patient’s quality of life, affect their mental health and impact their beliefs towards their medication [27]. Furthermore, in cases where side effects to medication are poorly tolerated, there is a risk that patients may skip doses or stop their medication entirely to cope [27]. In our study, most participants had a key treatment goal to improve their quality of life. We suggest that reducing medication burden through alternative treatment options and by working with patients to manage their medication regimen concerns could positively impact patients’ quality of life and, in turn, improve treatment adherence.

### Awareness and perceptions of renal denervation

The participants interviewed had a poor understanding of RDN as a treatment option for severe difficult-to-treat hypertension. This may reflect the limited access to this procedure in Australia [28].

A survey on patient preferences for RDN in Japan found that over 30% of patients with hypertension wanted to undergo RDN or would prefer RDN. Interestingly, this survey along with another conducted in Germany, found that as patients’ age increased their preference for RDN decreased suggesting that younger patients may be more motivated to avoid anti-hypertensive medication [29, 30]. Due to the small sample size, we were unable to determine preference for RDN by age. However, in our study, there was a strong desire to have RDN for severe difficult-to-treat hypertension, although some patients expressed concern about surgical risks and potential adverse effects such as hypotension. Patients in other studies have expressed these concerns about RDN, with the invasiveness of the procedure being the highest patient concern [31]. It was apparent in our study, that patients in Australia need more information on the procedure. HCPs are the main source of hypertension treatment information for patients in Europe, Japan, and the United States [29–31]. Furthermore, HCPs’ knowledge strongly influences patients’ decisions about medical treatment and studies have found that the main reason patients would choose RDN was if it was recommended by their doctor [30, 31]. Although RDN is a guideline recommended treatment for certain patients with severe difficult-to-treat hypertension [32, 33], it is not currently covered by Medicare. Participants in this study had limited awareness of RDN, which they attributed in part to their HCPs not raising it as an option, likely reflecting current funding constraints. Greater familiarity among both patients and clinicians may support more informed shared decision-making.

### Strengths and limitations

To our knowledge, this is the first study to assess treatment preferences and lived experiences of people with severe difficult-to-treat hypertension in Australia. Despite a relatively small sample size (*n* = 12), a recent review of qualitative research reported that studies reached data saturation at between 9 and 17 interviews [34]. Qualitative research offers valuable insight into patient perspectives [35]. Our study provides interpretative depth rather than generalized perspectives regarding experiences with severe difficult-to-treat hypertension specifically within the Australian healthcare system. Participants were recruited through convenience sampling, and mostly included metropolitan based individuals so the sample may not represent the wider Australian population with severe difficult-to-treat hypertension [36]. Additionally, it is possible that the proximal availability of Medicare safety nets to the participants may have influenced their clinical experiences. The findings in our study reflect the participants’ subjective insights and experiences rather than objectively verifiable behaviors, therefore, there is also a possibility of social desirability bias. The inclusion of two patients with lived experience of RDN strengthens our study. While understanding the RDN experience was not a primary objective of the study, it provides insight into the lived experience of RDN and how it supports or differs from perceptions of patients unaware of this option.

## Conclusion

While clinical trials provide valuable evidence on the safety and efficacy of therapy, they often overlook what matters to the patients receiving treatment, the burden of disease and impact on their quality of life. This study explores the experiences of people living with severe difficult-to-treat hypertension, their treatment goals and perceptions of RDN as a treatment option. Consistent with prior literature, this study highlights the significant physical, emotional and social impact of severe difficult-to-treat hypertension in Australia, as well as the clinical need for alternative treatment options. This study also highlighted the stigma experienced by some patients living with hypertension, particularly those diagnosed at a younger age; and instances of unsupervised self-titration of antihypertensive medication in response to alarming home blood pressure readings. Taken together with broader findings, these observations reinforce the need for clinical management of severe difficult-to-treat hypertension to extend beyond medication optimization and encompass psychosocial support and structured patient education on appropriate self-management in between consultations. Finally, participants in this study who had not received RDN, were unaware of RDN as a treatment option, reflecting its limited availability within the Australian public health system. This gap in awareness was identified as a barrier to informed patient-clinician discussion.

## Supplementary Information


Additional file 1: Table 1 - Interview Discussion Guide.



Additional file 2: Table 2 - Participant’s highest blood pressure reading.



Additional file 3: Table 3 - Participant quotes.


## Data Availability

Specific data supporting the conclusions of this article are included within the article and its additional files.
