# Supplementary material for: Qualitative insights into the experiences and goals of people living with severe difficult-to-treat hypertension in Australia
Source: BMC Cardiovasc Disord. 2026 Jun 19;26:752. doi: 10.1186/s12872-026-06141-9 (PMC13523477; doi:10.1186/s12872-026-06141-9)
Supplement: Supplementary file 1 [file 12872_2026_6141_MOESM1_ESM.docx]

# Additional File 1

## Table 1: Interview Discussion Guide

Research Objectives (for interviewer reference):

This qualitative research allows for an in-depth investigation of the whole experience within ‘difficult to control/treatment resistant hypertension’. This includes the impact on quality of life along with key treatment goals that matter to people when selecting a treatment. The feedback obtained from the qualitative interviews will be included in an MSAC submission for catheter-based renal denervation for a ‘high clinical need’ hypertension population (e.g., SBP ≥ 180 mmHg and/or DBP ≥ 120 mmHg, where optimal management with antihypertensive medication is not working).

The key research objectives for this qualitative phase are to:

Explore the patient journey within ‘difficult to control/treatment resistant hypertension’ from a holistic perspective including how people are impacted by the condition in their daily lives – this will include emotional, physical, financial, work, education, social factors, impact on family relationships etc.

Examine the treatment experiences and overall perceptions of treatment available within this therapy area. How do treatment benefits translate to real improvements in quality of life for people? And what do negative aspects of treatment mean for participants? Within this, understand patient perceptions of Renal Denervation Therapy (RDN).

What goals and outcomes are people hoping to achieve with treatments available for their condition?

*The aim of the qualitative discussion guide is to assist the flow of conversation during the interview. The questions will be used as a reference to ensure relevant topics/research objectives are covered comprehensively. The flow of conversation will be guided by participant responses and may not be asked in the order indicated below. Wording, phrasing and terminology will be guided by participant language. Topics that are raised spontaneously or in different sections of the guide will not be re-visited unless further information is required.*

| *Aim* | Discussion |
| --- | --- |
| *1. To explain purpose of research* | **Warm Up and Introduction** (5 min)   - *Thank participant for taking part in the research.* - *Explain purpose of the research:* Today I would like to talk to you about your high blood pressure, also known as hypertension, and how this condition has affected your life. I would also like to explore what your goals with treatment are. - *If questions are raised about current treatment:* If you are uncomfortable with any questions during the interview, let me know and we can move on to another question, or if you prefer, we can stop the interview at any time. We encourage you to contact your doctor if you have any questions about your high blood pressure or overall health. You can also contact a support group such as Hearts for Heart (Phone number: 1300 343 278) - *Explain anonymity.* Participants are guaranteed under Privacy Act and Association of Market and Social Research Organisations (AMSRO) code of conduct - findings from interviews will not be linked back to individuals. - *Explain obligation to report Adverse Events (AE):* If during the interview, you report a side effect, special situation or quality complaint that you have experienced while undergoing a treatment from the medical device company sponsoring this research, there is an obligation to report this complaint as part of the ongoing monitoring of their treatment. Information you provide during the interview relating to complaints will need to be emailed to the medical device company for their records. Please let me know if you are happy for me to do this?   INTERVIEWER NOTE: IF AE IS REPORTED, COLLECT INFORMATION AT THE END OF THE INTERVIEW AND CHECK IF THE PARTICIPANT PROVIDES PERMISSION FOR THEIR PERSONAL DETAILS TO BE INCLUDED OR IF THEY PREFER ANONYMOUS REPORTING   - *Length of interview*: Please note that discussion will take approximately 45-60 minutes. - *Payment:* There will be a payment of $120 for completing this interview, in the form of a gift card (physical or electronic) or you can choose to donate to advocacy. - *Medical Device company disclosure:* We will inform you of the name of the sponsoring company of this research at the end of this interview. - *Ask permission to record (for report-writing purposes).* |
| *2. Gain background information on patient, terminology used, and diagnosis* | **Participant Profile, Terminology, Diagnosis** (10 min)  Can I begin by asking you a few background questions?   - Age? - Work situation? Type of work? - Family/home-life (i.e. married, partner, single). - Location of where you live (i.e. state; metro, regional, rural)? - On a scale of 1-10, where would you rate your current overall level of health? PROBE: For what reason have you given this score?   CHECK PATIENT TERMINOLOGY AND USE FOR THE REMAINDER OF INTERVIEW:  Before we move on, I would just like to check how you refer to your condition?   - When you talk about your condition, how do you refer to it? (high blood pressure, hypertension?)   - With doctors? With friends/family? - What terminology do you use to explain that your condition remains uncontrolled (i.e., has not had sufficient response to medications you have tried?)   - ‘Difficult to control high blood pressure/hypertension?   - ‘Persistent hypertension’   - ‘Treatment resistant hypertension’ – have you heard of this term?     - Have doctors ever used it?     - Do you use it?     - What does this term mean to you?     - What is your understanding of what causes treatment resistance?       - High blood pressure readings (despite taking a combination of prescribed blood pressure lowering medication)?         - Are you aware of what these readings are? Are you aware of your current readings?         - Are you aware of how many medications this would include?       - Not taking medications as prescribed (i.e., non-adherence)?       - Other? - Aside from your ‘high blood pressure/hypertension’ do you have any other conditions for which you are receiving treatment? IF SO: Which conditions? - Do you have a family history of ‘high blood pressure/hypertension’?   IF SO:   - Who else in your family has this condition? To what extent have they been sufficiently controlled with medications (i.e., treatment resistance)?   And now thinking now about your diagnosis:   - When were you diagnosed with your initial ‘high blood pressure/hypertension’? - What were the events that led to the diagnosis? Were you experiencing symptoms at the time? IF SO: What type?   - What type of doctor diagnosed the initial ‘high blood pressure /hypertension’? - How long after the initial diagnosis did it become ‘difficult to control/treatment resistant’? - How was this established?   - By the number of medications needed to lower your blood pressure?     - What is the maximum number of blood pressure lowering medications you have been on at the same time?     - How many are you currently taking? INTERVIEWER: NOTE FOR LATER DISCUSSION     - Are you aware if any of the tablets you have/are taking include combinations of medicines in the one tablet? IF SO: Which products?       - Are you aware of the different doses of the various medications within the combination tablet?     - How else? (e.g., doctor examination, medical history, blood pressure readings, other tests etc?)   - What doctor type established this? (i.e. did the initial doctor refer you to another type of doctor)? What did this doctor tell you about it at the time? |
| *3. Identify the impact of treatment resistant hypertension (including adherence to medication); explore role of carer* | **Impact of Condition, Adherence to medication and Role of carer (15 min)**  I would now like to ask what the impact of ‘difficult to control/treatment resistant hypertension’ has been on life.   - Can you summarise how this condition has affected you? SPONTANEOUS   - Physical impact?   - Health implications?     - Symptoms? What type? PROBE:       - How have these affected you? Please provide examples.       - How long do symptoms last for? Do they come and go? Do they vary in severity?     - Related conditions/complications? What type? (e.g., kidney damage, stroke, cardiovascular etc)       - How have these affected you? Please provide examples.       - Are you aware if these conditions have been directly linked to your ‘difficult to control/treatment resistant hypertension’ by your doctor?         - What did the doctor say about this?   - Lifestyle changes? (e.g., diet, exercise, smoking, drinking)? What impact has this had on you?   - Side effects with treatment? What type? How long did these last for? How do you know they were related to the treatment you were taking? How have these affected you? (BE AWARE OF RELEVANT AE’s)   - Have you ever spent time in hospital because of your ‘difficult to control/treatment resistant hypertension’?   IF YES:   - For what reasons? - How often? - Did you present to emergency or have to call an ambulance? - How long were your hospital stays? - What impact did spending time in hospital have on you?   - Logistical factors? - E.g. Travelling to and from hospital/appointments? How far do you have to travel? What has been the impact of this for you?   FOR REGIONAL BASED/RURAL PARTICIPANTS:   - How if at all, do you feel that your management of you ‘high blood pressure/hypertension’ has been impacted because of where you live? Any issues encountered? - Being able to attend to your daily activities (e.g., responsibilities, duties)? What type?   - Medication/adherence? PROBE FULLY   CONFIRM HOW MANY MEDS PARTICIPANT IS CURRENTLY TAKING:   - You mentioned earlier that you are currently taking XX to treat your ‘high blood pressure/hypertension’? How do you feel about this?   - And for any other conditions? What is the total number of medications you are taking each day?   - What impact does your medication load (number of medications you need to take each day) have on your quality of life?   - Have there ever been circumstances where you haven’t taken your medications as prescribed? For what reasons?     - Forgetting to take? Missed doses?       - Under what type of circumstances?     - Not sure how to take?     - Decided not to take? For what reason?       - High burden of being on multiple medications? How so?       - Scheduling of medications? How many times a day?       - Difficulty swallowing tablets?       - Don’t believe they are effective?       - No longer symptomatic?       - Side effects?       - Insufficient support provided? By whom?       - Cost?   - Have you ever decided to stop medication(s) altogether? For what reasons?   - Have doctors discussed the importance of taking medications as prescribed/recommended for your ‘difficult to control/treatment resistant hypertension?   IF YES:   - What did they say about this? - How important would it be for you to be able to reduce the number of medications that you take? Why do you say that? What would it mean for your quality of life? - Other physical impacts/restrictions? - Emotional impact? - Mental health? (e.g. anxiety, depression?) - Concern/uncertainty? (e.g., future complications? What type? Long-term effects of medication) - Frustration? e.g., Medication load? Remaining uncontrolled with medication (i.e., medication is not effective enough)? Other? - Social impact? (impact on friendships? social activities?) - Financial impact? - What type of costs have incurred because of your ‘difficult to control/treatment resistant hypertension’? Have these been on-going or one-off costs? E.g. specialist appointments, travel etc? - Do you have private health insurance? How has this helped, if at all? - Impact on work/education? - Reduced capacity? (i.e. reduced hours/inability to work). What has this meant for you? - Impact on family? - IF RELEVANT: Ability to support your family (e.g. children, grandchildren, elderly parents)? How so? - Family relationships? - Day to day interactions with family? How so? - Family holidays?   ***Role of carer***   - Do you have a key person who helps you with your ‘difficult to control/treatment resistant hypertension’? - Who, and how do they help? (i.e. spouse, partner, paid/unpaid carer) - What is the impact of your condition on them? POTENTIAL PROBES: physical, emotional, social, financial? |
| *4. Obtain information on role of HCPs, treatment satisfaction and experience* | **HCP, Satisfaction and Treatment Experience** (15 mins)  Before discussing treatment, I’d like to ask you about the overall role of doctors involved in the management of your ‘difficult to control/treatment resistant hypertension’ and also your overall satisfaction with treatment received?   - Role of GP? i.e., diagnosis, prescribing treatment? referral (to what type of specialist)? Ongoing monitoring? (what type and how often) - Role of specialist? i.e., prescribing treatment? monitoring? (what type and how often?) - To what extent have you been involved in treatment decisions? - To what extent have you been satisfied with the treatment received? On a scale of 1-10 where 1 is not at all satisfied and 10 is extremely satisfied? Why do you say that?   - What do you feel are areas for improvement, if any?   I’d now like to obtain a summary from you regarding treatment you have received.  MEDICATION DISCUSSION: OBTAIN A HISTORY OF MEDICATION TAKEN. CAPTURE EXPERIENCES FOR DIFFERENT STAGES. TALK GENERALLY IF PARTICIPANT IS NOT AWARE OF SPECIFIC TREATMENT RECEIVED.  Can you take me through your treatment journey starting from the first medication(s) prescribed to where you are at now? If you can remember, please let me know the: PROBE ON: initial, during medication journey, current medication.  INITIAL MEDICATION   - Name of medication(s)? - How taken (i.e. alone or in combination) - Why were these prescribed at this stage? What did your doctor say?   IF RELEVANT:   - Reasons for switching?   REPEAT ABOVE QUESTIONS FOR DURING JOURNEY AND CURRENT MEDICATION  Thinking about the timing of medications:   - Approximately, how long did it take to go from one to two medications? - And then to three or more medications?   - Did this combination work initially?   - At what point did your blood pressure not go down despite being on three or more medications?   Thinking about overall benefits and challenges with medication:   - How have these medications provided benefits (i.e., positive impact on your quality of life)? - What have been the challenges? BE AWARE OF AEs   IF NOT COVERED PREVIOUSLY:   - Side effects? How long did these last for? - Medications interacting with each other? - Medications not suitable for comorbidities? - Medications not sufficiently effective? - Not being able to tolerate medication for long?   SURGERY DISCUSSION   - Has your doctor ever discussed non medication options for your condition? I.e., surgery? - Have you received surgery for your ‘difficult to control/treatment resistant hypertension’?   IF YES:   - Type of surgery? IF RDN ASK:   - What type of doctor recommended this? (i.e. GP, cardiologist?)     - What led them to make this recommendation?       - Already on 3 or more medications?       - Due to comorbidities? Which in particular?       - Higher risk of side effects? For what reasons? (e.g., age)   - How long did you have to wait for this surgery?   - How long were you in hospital for?   - What was involved?   - What did it cost?   - How did you feel after surgery? How long did it take you to recover?   - How do you feel in the long run after having had this surgery?     - What have been the benefits? How has your quality of life been improved (if at all)?       - Has this surgery decreased the need for medication? How so? Thoughts on this?     - What have been the challenges (if any)?   IF NO:  Have you heard of a surgical intervention called renal denervation (or renal ablation)?  IF YES:   - What do you know about this surgery? - What would be the benefits/challenges of this surgery in your opinion? - Is renal denervation something you would be willing to consider for treatment resistant hypertension? Why/why not?   READ DESCRIPTION BELOW TO ALL PARTICIPANTS:  I would like to read a description to you about renal denervation (RDN) and obtain your thoughts on this.  *Renal Denervation is an innovative, minimally invasive procedure. It is a single procedure that has an ongoing blood pressure effect. It works by targeting specific nerves near the kidneys that can become overactive and cause high blood pressure. During the procedure, an interventional cardiologist uses a catheter to send energy (ultrasound or radiofrequency energy) to the overactive nerves near the kidneys to decrease their activity and therefore decrease your blood pressure. The procedure lasts approximately an hour and an overnight stay may be required following the procedure.*  *The effects of the renal denervation procedure work 24 hours a day. This treatment may not be a replacement for having to take medication, however, it could help lower your blood pressure and if so, may allow you to reduce the amount of blood pressure medications (either the number and/or dosage) that you need to take (depending on your response and health status). It may also help reduce complications of your condition such as cardiovascular disease, renal disease, heart failure, strokes, atrial fibrillation and it can improve overall quality of life.*  IF PARTICIPANT HAS NO EXPERIENCE WITH RDN   - What are your thoughts on this option? - What would be the benefits?   - One off procedure?   - Improvement in lowering blood pressure?   - Potentially being able to reduce the number and/or dosage of required medication,   - Potentially reduce other complications of high blood pressure (e.g., cardiovascular disease etc)?   - Permanent effect? - What would you be worried about? (i.e. barriers)?   POTENTIAL PROBES: costs, side effects, risks with surgery (what type?), reluctance to stay in hospital overnight, does not treat co-morbidities contributing to condition.   - Is renal denervation something you would be willing to consider for your ‘difficult to control/treatment resistant hypertension? Why/why not?   - What would encourage you to consider? (e.g., doctor recommendation?) - What further information would you like to know about this option?   IF PARTICIPANT HAS EXPERIENCE WITH RDN   - How well does this description fit with your own personal experiences/perceptions of renal denervation? How so? - Can you summarise the key benefits? Experiences with:   - Improvement in lowering blood pressure?   - Reducing the number and/or dosage of required medication? What has this meant for you?   - Reducing other complications of high blood pressure/hypertension (e.g., cardiovascular disease etc)?   - Permanent effect? - Challenges?   - Surgery? Risks? Costs?   ASK ALL/PROBE FULLY:   - To what extent do you feel there is a need to have this option available in Australia for people living with ‘difficult to control/treatment resistant hypertension? Why do you say that? ‘   - Are there any patient types who would be more suitable for this option? Why these? - What further information would you like to know about this option? |
| *5. Explore treatment goals* | **Goals with Treatment Resistant Hypertension Treatment** (15 min)  In this last section, I would like to ask you about the key outcomes with treatment you are hoping to achieve.   - What are your key goals with treatment? - What changes would you like to see happen as a result of taking treatment? - How would achieving these goals impact on your life?   PROBE ON   - What are your physical goals? How can treatment help you achieve these goals? - What are your emotional goals? How can treatment help you achieve these goals? - What are your social goals? How can treatment help you achieve these goals? - What are your work goals? How can treatment help you achieve these goals? - What are your financial goals? How can treatment help you achieve these goals? - What are your family goals? How can treatment help you achieve these goals? - Other goals?   IF RELEVANT: How would achieving these goals impact on your carer’s life?  In terms of the treatment,   - To what extent do you feel medication for your ‘difficult to control/treatment resistant hypertension’ can help you achieve these goals? - To what extent do you feel renal denervation can help you achieve these goals?   PROBE FULLY: physical, emotional, social, work, financial, family goals?  That brings us to the end of our discussion. Thank you for taking the time to participate – it helps with understanding how people manage ‘difficult to control/treatment resistant hypertension’ and their perceptions of treatments available for the condition. Is there anything else that you would like to add today? This research has been sponsored by Medtronic.  ORGANISE PAYMENT |

## Table 2 Consolidated criteria for reporting qualitative studies (COREQ): 32-item checklist

| **No** | **Item** | **Guide questions/description** | **Page addressed** |
| --- | --- | --- | --- |
|  | **Domain 1: Research team and reflexivity** | |  |
|  | **Personal Characteristics** | | |
| 1. | Interviewer/facilitator | Which author/s conducted the interview or focus group? | 5 |
| 2. | Credentials | What were the researcher’s credentials? E.g. PhD, MD | 5 |
| 3. | Occupation | What was their occupation at the time of the study? | 5 |
| 4. | Gender | Was the researcher male or female? | 5 |
| 5. | Experience and training | What experience or training did the researcher have? | 5 |
|  | **Relationship with participants** | | |
| 6. | Relationship established | Was a relationship established prior to study commencement? | 5 |
| 7. | Participant knowledge of the interviewer | What did the participants know about the researcher? e.g. personal goals, reasons for doing the research | 5 |
| 8. | Interviewer characteristics | What characteristics were reported about the interviewer/facilitator? e.g. Bias, assumptions, reasons and interests in the research topic | 5 |
|  | **Domain 2: study design** | | |
|  | **Theoretical framework** | | |
| 9. | Methodological orientation and Theory | What methodological orientation was stated to underpin the study? e.g. grounded theory, discourse analysis, ethnography, phenomenology, content analysis | 7 |
|  | **Participant selection** | | |
| 10. | Sampling | How were participants selected? e.g. purposive, convenience, consecutive, snowball | 4 |
| 11. | Method of approach | How were participants approached? e.g. face-to-face, telephone, mail, email | 4 |
| 12. | Sample size | How many participants were in the study? | 6 |
| 13. | Non-participation | How many people refused to participate or dropped out? Reasons? | - |
|  | **Setting** | | |
| 14. | Setting of data collection | Where was the data collected? e.g. home, clinic, workplace | 5 |
| 15. | Presence of non-participants | Was anyone else present besides the participants and researchers? | 6 |
| 16. | Description of sample | What are the important characteristics of the sample? e.g. demographic data, date | 6 |
|  | **Data collection** | | |
| 17. | Interview guide | Were questions, prompts, guides provided by the authors? Was it pilot tested? | 5 |
| 18. | Repeat interviews | Were repeat interviews carried out? If yes, how many? | No |
| 19. | Audio/visual recording | Did the research use audio or visual recording to collect the data? | 5 |
| 20. | Field notes | Were field notes made during and/or after the interview or focus group? | 7 |
| 21. | Duration | What was the duration of the interviews or focus group? | 5 |
| 22. | Data saturation | Was data saturation discussed? | 5 |
| 23. | Transcripts returned | Were transcripts returned to participants for comment and/or correction? | 5 |
|  | **Domain 3: analysis and findings** | | |
|  | **Data analysis** | | |
| 24. | Number of data coders | How many data coders coded the data? | 5 & 6 |
| 25. | Description of the coding tree | Did authors provide a description of the coding tree? | 9 |
| 26. | Derivation of themes | Were themes identified in advance or derived from the data? | 5 & 6 |
| 27. | Software | What software, if applicable, was used to manage the data? | 6 |
| 28. | Participant checking | Did participants provide feedback on the findings? | 5 |
|  | **Reporting** | | |
| 29. | Quotations presented | Were participant quotations presented to illustrate the themes / findings? Was each quotation identified? e.g. participant number | 6-25 |
| 30. | Data and findings consistent | Was there consistency between the data presented and the findings? | 9-31 |
| 31. | Clarity of major themes | Were major themes clearly presented in the findings? | 9 |
| 32. | Clarity of minor themes | Is there a description of diverse cases or discussion of minor themes? | Table 2 - 26 |
