# Supplementary material for: Qualitative insights into the experiences and goals of people living with severe difficult-to-treat hypertension in Australia
Source: BMC Cardiovasc Disord. 2026 Jun 19;26:752. doi: 10.1186/s12872-026-06141-9 (PMC13523477; doi:10.1186/s12872-026-06141-9)
Supplement: Supplementary file 2 [file 12872_2026_6141_MOESM2_ESM.docx]

Additional File 2

| **ID** | **Highest Reading** |
| --- | --- |
| P1 | 228mmHg/ 125mmHg |
| P2 | 205mmHg/ 90mmHg |
| P3 | 190mmHg/ 110mm Hg |
| P4 | 180mmHg/ 90mmHg |
| P5 | 180mmHg/ 110mmHg |
| P6 | 210mmHg/ 140mmHg |
| P7 | 220mmHg/ 160mmHg |
| P8 | 170 mmHg/ 121mmHg |
| P9 | 220 mmHg/ 115mmHg |
| P10 | 177mmHg/ 145mmHg |
| P11 (RDN/ 2020) | 240mmHg/130mmHg |
| P12 (RDN/2023) | 220mmHg/ 200mmHg |

Table 2: Participant’s highest blood pressure readings
