# Supplementary material for: Qualitative insights into the experiences and goals of people living with severe difficult-to-treat hypertension in Australia
Source: BMC Cardiovasc Disord. 2026 Jun 19;26:752. doi: 10.1186/s12872-026-06141-9 (PMC13523477; doi:10.1186/s12872-026-06141-9)
Supplement: Supplementary file 3 [file 12872_2026_6141_MOESM3_ESM.docx]

**Additional file – Table 3**

| **Themes** | | **Participant quotes** |
| --- | --- | --- |
| **Diagnosis and monitoring of hypertension** | **Diagnosis and monitoring of hypertension** | “I kept feeling this weird giddiness, dizziness, so I went to the doctor, and he took my blood pressure.” P7 |
|  |  | “I was first told I had high blood pressure at the blood bank, and they said my blood pressure was too high for them to take [my blood], it was unsafe. That was in the 1980s.” P4 |
|  |  | “I had what they called a TIA [transient ischemic attack], […] then I had all these tests and that’s when they threw all these tablets at me.” P1 |
|  |  | “For several months I had to take regular blood pressure readings, keep a record of that, and then I was regularly seeing the doctor…he was trying me on various medications and then we arrived at what worked.” P8 |
|  | **Severe difficult-to-treat hypertension** | “For some reason the tablets weren’t working later on. I don’t know what happened, but the readings just exploded.” P10 |
|  |  | “Over time it has sort of been controlled and then all of a sudden, it’s burst out again. There’s been different types of drugs, some of which I had adverse reactions to, others which seemed to work, and it’s been going on now really, pretty much constantly ever since….to me it’s erratic, it’s hard to control.” P2 |
|  |  | “My GP has tried every drug on the planet and then you get one that makes you pee a lot which I didn’t like. So, it was a matter of experimenting with so many, I can’t remember all the names but just trying them all.” P7 |
|  |  | “If I have an anesthetic, it spikes really high, and…the anesthetist has to control it.” P2 |
|  |  | “I went in with this split aortic aneurism and then from there they discovered the high blood pressure…..I’m not really sure if it started before that….it’s been very difficult to control post that.” P3 |
| Impact of severe difficult-to-treat hypertension on quality of life | Physical impact | “When it went really low, I mean I couldn’t even stand up out of the chair. I thought I was going to faint and pass out. Blood pressure issues do have a big impact on my life.” P1 |
|  |  | “Dizziness, so making it too low, visual disturbances. I get quite, I can’t see.” P3 |
|  | **Emotional impact** | “I do get emotional, I get very teary, and I try to hold it back.” P1 |
|  |  | “It’s the impact on your psyche, like God, this is going to shorten my life because it just can’t be fixed, and that’s the fear factor.” P2 |
|  |  | “In about 2016 my blood pressure was something like 240/130. I did not record the date or time, and stopped monitoring for some time after that, as I was expecting a tap on the shoulder from the grim reaper at any time.” P11 |
|  |  | “Then I think, is one of these episodes going to lead to a stroke …. especially when you live alone.” P1 |
|  |  | “Because if I’m high risk and I’m by myself, nobody will find me, you know. You think about it from time to time. […] nobody is going to call 000 except me…nobody is going to find me. So yeah, that worries me a bit.” P10 |
|  | **Impact on family** | “No, I don’t think it’s affected him. He just has to take me to the GP or go and fill the script.” P5 |
|  |  | “Whether they’re going to get it, whether it’s going to be passed down to them and more than likely it will be…I don’t know what they’re thinking about it. One of them said, ‘Oh that’s great, thanks!’” P9 |
|  | **Social impact** | “I miss bushwalking because I can’t now, so I’ve got a mobility scooter…for people going on trails...It has impacted having fun.” P7 |
|  |  | “I don’t consume a lot of salt….and I’m not fat....I stay active, I ride my push bike to and from work and […] I do a lot of hiking.” P11 |
|  |  | “I lost weight, try to eat better…a bit more exercise…and probably sleeping more.” P3 |
|  |  | “Yeah in the evening I feel a bit “blear”. Is that what the word is? Dizzy with the blood flow. I can feel it wearing off. And so part of my church, we have a life group so I find it really hard […] to go when they have it in the evening because of these nevus in my eyes. I don’t like driving because it’s hard to see. But also, it is wearing off and it makes me very tired and I hate sitting there yawning. So, tiredness is really a side effect too.” P7 |
|  | **Financial impact** | “I mean if you add up [the] money I spent on medication over the years, it would be probably significant.” P6 |
|  | **Work impact** | “Well, my work capacity has altered but.…I wouldn’t say it’s the blood pressure; I think it was the other thing [aortic aneurysms].” P3 |
| **Management of severe difficult-to-treat hypertension** | **Role of health care practitioners** | “I think mainly it’s been the doctor’s responsibility. I’ve basically let him work it out except of course if I’ve got a side effect and of course reporting it and he’s changed the medication straightaway. But the decision on what to change to has always been his.” P3 |
|  |  | “I went through all kinds of tests and treadmill tests and the whole thing. [I’m] not seeing any specialists at the moment.” P7 |
|  | **Experiences with antihypertensive medication** | “Overall, it’s okay, I just find sometimes it’s, I like it within a parameter and sometimes it’s above and I think well, I’ve done nothing, I take the medication, why is it above?” P10 |
|  | **Current medications** | “I think I was on just that one for probably ten years…And then that’s when I went to the doctor and they […] put me on maybe two types and that worked for maybe […] one to two years and then…since I’ve been on these three combined, it’s generally been pretty stable.” P6 |
|  |  | “I think it was two and then it changed about eight times. I could see even in that time, it’s evolved, new medications have come out, like this one and the combinations.” P9 |
|  | **Key benefits and challenges with antihypertensive medication** | “Keeping you alive, getting your blood pressure down to a more, I suppose manageable level, life-saving, life improvement.” P7 |
|  |  | “If I don’t keep up with the meds it goes through the roof again, it goes up and down like a yo-yo. Now it’s stable, going down instead of going up and down.” P4 |
|  |  | “What I’d worry about…what other options are there for me?...just the thing of well, if the next one doesn’t work what do I do, and then it’s resistant to that. Like when does it end, like what will work? If this [current medication] starts resisting I’d like to think or certainly hope that there would be some other options or combinations of medication or something in the future that can more directly treat high blood pressure. That becomes more of a concern as I get older in terms of stroke and heart attack, all these different things that can occur, that’s on my mind.” P6 |
|  |  | “The medicine I’m on...it’s really hard to manage. I can feel when it’s off because of that giddy feeling that you get...The sleepiness and the dizziness and the side effects of the medication.” P7 |
|  | **Medication adherence** | “Yeah, they have, they basically said it’s keeping you alive.” P10 |
|  |  | “Well, they just said, you know, follow the directions, take it consistently.” P6 |
|  |  | “I quite often forget the morning ones and then realize it’s 11 o'clock or 12 o'clock and I haven’t taken my medication.” P9 |
|  |  | “Yes, only a couple of days ago, I went, ‘Did I take the medication or didn’t I?’ I couldn’t remember and I thought, ‘Oh I probably did.’ And I don’t know the answer, I thought, ‘Stuff it, it’s too late now I’ve got to go to sleep.’” P10 |
|  |  | “I wonder how that’s going to go, the older I get. People, you know how they start to get a bit forgetful and it’s a lot to remember.” P2 |
|  | **Medication load** | “[I feel] despondent, I don’t like it… every time I have to look at the little plate with all the tablets on it, I think, my God, you know, it’s so many and I don’t like it but I’ve got to do it, but it makes me unhappy.” P2 |
|  |  | “I don’t feel healthy because I’m taking all these tablets and I shouldn’t be taking the tablets, but I know that I’ve got problems in the future that I can’t solve and the tablets are managing it for me because my body can’t manage it. So, it makes you feel a bit uneasy.” P10 |
|  |  | “At first it was pretty confronting but now I’m pretty used to it […] just took me a little bit to adjust to that I suppose.” P3 |
|  |  | “Maybe I’m a bit over it yeah. Well, it is sort of, I don’t know, I can’t think of any words, it identifies me as this, like even when my grandkids come around, ‘Oh all these tablets nan.’” P1 |
|  |  | “In the morning, I will take seven, and in the evening, I take four and then painkillers in-between.” P5 |
|  |  | “I take eight in [the] morning, two at lunch and nine at night.” P12 |
|  | **Treatment goals** | “To keep normal blood pressure…well it means that I can still work, exercise, participate in activities of daily living.” P3 |
|  |  | “Something that is going to keep it controlled and you know, keep me alive longer, reduce the risk of things like heart attack and stroke and cardiovascular situations no matter what the treatment is, that would be my goal. And for it to be as non-invasive as possible, and as reasonably convenient as possible.” P6 |
|  | **Treatment goal – reducing medication load** | “My aim is to get off all of them and, which is what I’m working through at the moment with my GP to just build up my own health […]. I’d rather not take any if I could get away with it. However, the reality of being 68, I may have to stay on one or two of them, but I want to eliminate as many as possible.” P8 |
|  |  | “I’m hoping to achieve the best possible health I can maintain at my age. That’s about it really, get off medicating.” P8 |
|  |  | “Maybe there is something else going on that needs to be fixed within my heart or whatever, and then I won’t need the tablets.” P1 |
| **Awareness and perceptions of renal denervation** | **Awareness and perceptions of renal denervation** | “No. That’s never been discussed, I don’t know about any surgery.” P1 |
|  |  | “I think innovative ways of treating things are incredibly important.” P6 |
|  |  | “The more options the better absolutely, I’d welcome it, at least it gives me something to think about, that’s helping me health-wise.” P10 |
|  | **Perceived benefits of renal denervation** | “Well, it means I would get back into performing as a musician as well as all the other things I used to enjoy doing. Yeah, I’d be more active, that’s got to be a plus. […] Just for much better quality of life.” P8 |
|  |  | “I guess the thought that a few things have been fixed or potentially fixed. I reckon that’s probably a psychological positive.” P2 |
|  |  | “[It would] take away a very severe burden, something that’s in the back of your mind worrying you, which puts our blood pressure up.” P7 |
|  |  | “If the medication became less, it would be a total bonus as well.” P2 |
|  |  | “Well, I think that any medication has got side effects and probably a lot we don’t know, so if your body can be healed or fixed in a way that it doesn’t…falsely put something into it, I’d say that that’s a good thing.” P3 |
|  |  | “This is vital, this is going to save the country money because at the very least you are reducing the medication, so people’s health is going to go up […]. It’s a win/win… it’s actually very innovative and if you can lower the pills or stop them altogether, I would sign up for that. As I said, I don’t like taking medication I don’t think it’s good for your body… take once, that’s exactly what I’m after, just better than a pill.” P7 |
|  |  | “Look, a one-off sounds nice if it wasn’t painful.” P3 |
|  | **Perceived challenges of renal denervation** | “I think it’s pretty invasive...There is always risks involved isn’t there in surgery? Damage to the nerve and I suppose bleeding, and your general anesthetic risks.” P5 |
|  |  | “If you’ve got to take one tablet, what difference does it make if you’ve got to take two? I personally wouldn’t try it because in that description you are saying that you could possibly reduce your blood pressure medication, they didn’t say that you would be able to not take them.” P5 |
|  | **Renal denervation as a treatment option** | “The tablets should work better, so I would rather say, well okay, bring on a new procedure. It sounds good. Where do I get that done?” P1 |
|  |  | “I’m not sure I’d want to have it done. I mean I get anxious going under the knife, so I try to avoid having any sort of surgery unless it’s really necessary.” P5 |
|  |  | “You wouldn’t have side effects of medication would you, and hopefully it would help you avoid having strokes and cardiovascular things.” P5 |
|  |  | “Because it’s going to the root cause […] and gives you a result that, it’s the magic potion, it’s just brilliant.” P7 |
|  |  | “Well decreasing amounts of medication to maintain a healthy blood pressure is what I’m aiming for. I mean I may not be able to get rid of all of my medications but if I can reduce them, that’s great. It’s fantastic.” P8 |
|  | **Further information on renal denervation** | “It sounds a little scary. I suppose you want to know more. I suppose it’s sort of exciting too if it has benefits.” P3 |
|  |  | “I mean obviously no one wants to be a guinea pig and they just want a few people to see how it’s going, but as long as it’s already been proven and it works….I think it sounds good.” P1 |
|  |  | “It sounds like I’m having to all of a sudden do something on top of what I’m already doing which, you know, taking time off work to do that, having to go overnight in hospital, that sounds more concerning to me than taking medicine…. But if there was a lot of evidence and research showing that it is safe and it does work and there is a 95% chance you won’t need medication anymore then I would be more likely to consider it.” P6 |
|  |  | “I’d have a good look at what it is, what the pros and cons of it are, weigh the benefits against the disadvantages of it. […] If I was given all the relevant information about it, I would consider, but just going in blindly, no.” P4 |
